# AFragmenter: schema-free, tuneable protein domain segmentation for AlphaFold protein structures

**DOI:** 10.1093/bioinformatics/btaf588

**Published:** 2025-10-27

**Authors:** Stefaan Verwimp, Rob Lavigne, Cédric Lood, Vera van Noort

**Affiliations:** Department of Microbial and Molecular Systems, KU Leuven, Leuven 3001, Belgium; Department of Biosystems, KU Leuven, Leuven 3001, Belgium; Department of Biosystems, KU Leuven, Leuven 3001, Belgium; Department of Biology, University of Oxford, Oxford OX1 3SZ, United Kingdom; Department of Microbial and Molecular Systems, KU Leuven, Leuven 3001, Belgium; Institute of Biology Leiden, Leiden University, Leiden 2333 BE, The Netherlands

## Abstract

**Summary:**

Protein domain segmentation is a crucial aspect of understanding protein functions and interactions, and it is vital for protein modelling exercises and evolutionary studies. Current segmentation methods often rely on predefined classification schemes, leading to inconsistencies and biases. AFragmenter provides a schema-free and tuneable approach to protein domain segmentation based on network analysis of AlphaFold-predicted structures. Utilizing Predicted Aligned Error values, AFragmenter constructs a fully connected network of protein residues and identifies distinct structural domains by using Leiden clustering. This method empowers users to adjust parameters including contrast threshold and resolution, providing control over the segmentation process.

**Availability and implementation:**

AFragmenter is implemented in Python3 and freely available under an MIT license. It can be found as a Python library and command line tool at https://github.com/sverwimp/AFragmenter, pip, and Conda.

## 1 Introduction

Protein domains are generally defined as self-stabilizing regions that can support independent biological functions. However, this definition only captures one of several perspectives, as partitioning protein structures into domains can be based on several criteria, such as evolutionary conservated motifs, dynamically coupled segments, or spatial separation (Dawson *et al.* 2017, Postic *et al.* 2017). Regardless of the framing, delineating these domains is important to studying their functions and supporting applications such as enzyme engineering, drug design, and comparative genomics (Poleszak *et al.* 2012, Tong *et al.* 2022).

This multi-criteria nature of domain definition has resulted in discrepancies in domain assignment across different studies, as researchers may interpret structural features and functional roles differently (Taylor 1999, Veretnik *et al.* 2004). These inconsistencies have propagated into the various domain databases, where identical queries return different results. For example, MET8 (UniProt: P15807, PDB: 1KYQ) is classified as a three-domain protein in both CATH (Knudsen and Wiuf 2010) and ECOD (Cheng *et al.* 2014). In contrast, SCOPe (Chandonia *et al.* 2022) categorizes it as a two-domain protein, while InterPro (Paysan-Lafosse *et al.* 2023) contains two domains but also includes an additional unintegrated Pfam domain (Mistry *et al.* 2021). Meanwhile, SCOP (Lo Conte *et al.* 2000) considers MET8 to be a single-domain protein. Such discrepancies across classification systems highlight the inherent complexity of defining protein domains. Real-world protein structures often exhibit diverse and irregular domain architectures, including discontinuous domains that are not contiguous in the amino acid sequence (Dawson *et al.* 2017, Wang *et al.* 2021). These complexities make domain segmentation, the process of identifying and separating protein domains, a particularly challenging task.

Efforts have been made to create and improve domain segmentation tools to alleviate manual work from researchers. At least fourteen new or improved methods have been published between 2019 and 2024 (Hong *et al.* 2019, Jiang *et al.* 2019, Shi *et al.* 2019, Eguchi and Huang 2020, Zheng *et al.* 2020, Mulnaes *et al.* 2021, Cretin *et al.* 2022, Mahmud *et al.* 2022, Wang *et al.* 2022, Lau *et al.* 2023, Yu *et al.* 2023, Zhang *et al.* 2023, Zhu *et al.* 2023, Wells *et al.* 2024). Among the newly published methods, ten out of fourteen are machine-learning based. Merizo (Lau *et al.* 2023) and Chainsaw (Wells *et al.* 2024) are two such methods and are among the best performing domain segmentation tools available, at the time of writing this paper, based on benchmarking results against data from CATH. The absence of definite standards on protein domains can lead to machine-learning based approaches to incorporate a bias towards the domain classification scheme used by their training data. Similarly, heuristic methods frequently suffer from this issue if their methods were fine-tuned using this same data. A second issue with the aforementioned referenced domain segmentation methods is their lack of flexibility (see Table 1, available as supplementary data at *Bioinformatics* online). These tools do not allow the user to significantly influence the segmentation process of the protein structure. Although this inflexibility may not always pose a major problem, it can render these tools impractical when their results do not align with researchers’ interpretations or appear illogical.

To address the bias towards certain domain segmentation schemes and the lack of flexibility, we developed a schema-free, flexible protein segmentation method, based on network clustering of AlphaFold structures. Our approach leverages AlphaFold’s Predicted Aligned Error values, which captures predicted confidence in inter-residue alignment and thus provide a basis for identifying independent structural units within a protein. This method does not rely on any predefined classification scheme and allows users to fine-tune a limited set of parameters, enabling them to explore multiple partitionings of a protein structure. Our method aims to strike a balance between performance, user control, and ease of use.

## 2 Implementation

AFragmenter is an AlphaFold structure segmentation tool based on a network clustering approach. It constructs a fully connected network where the nodes represent the residues of a protein, and the weights are derived from the Predicted Aligned Error (PAE) values from AlphaFold. PAE values measure the estimated error of inter-residue distances, reflecting the confidence of the model in relative residue positions. High PAE between segments indicates lower confidence in their relative arrangement, suggesting they may behave as independent entities or flexible linkers. Conversely, low PAE signifies high confidence in a self-contained structural unit (Jumper *et al.* 2021, McCafferty *et al.* 2023, Roca-Martínez *et al.* 2024, Varadi *et al.* 2024). Recent studies also indicate AlphaFold models, including PAE, implicitly capture protein dynamics (Guo *et al.* 2022), a property frequently associated with distinct domain movements. Consequently, intra-domain residue pairs are expected to have lower PAE values compared to inter-domain pairs. This difference distinguishes well-structured regions within a protein structure. By using these values as edge weights for a network and applying Leiden clustering, AFragmenter clusters protein residue pairs of well-structured regions together, thus achieving protein structure segmentation.

### 2.1 Creation of the protein structure network

**Figure 1.**
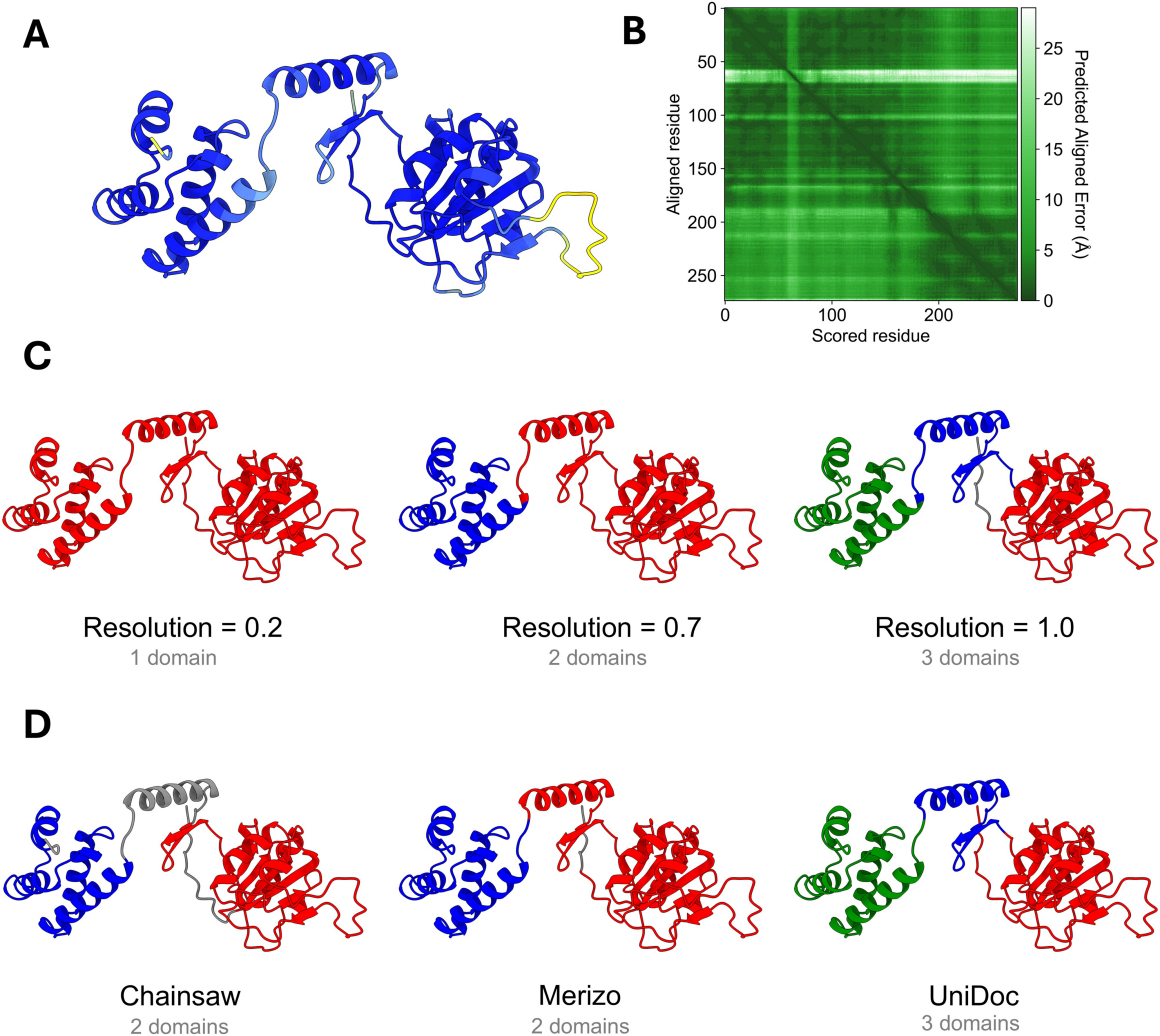
(A) The predicted AlphaFold protein structure of MET8 (P15807). Colours correspond to the pLDDT score values; dark blue indicates high confidence regions, while lighter colours represent lower confidence regions. (B) The PAE plot of MET8. One noticeable band of high PAE scores corresponds to the disordered segment with low pLDDT scores. (C) Segmentation results achieved by tuning the resolution parameter of AFragmenter (other parameters default). (D) A comparison of different domain segmentation methods for the AlphaFold structure of MET8. Domains are coloured red, blue, and green. Grey indicates residues that are not assigned to any domain.

The python interface to the igraph library (Csárdi and Nepusz 2006) is used to construct the protein structure network and to perform Leiden clustering. Each residue of the protein structure is represented as a single node in the network, and the edges between these nodes are weighted based on PAE values. The weight between nodes *i* and *j* is calculated as follows: $w_{ij}=\frac{1}{1+e^{\left(PAE_{ij}-T\right)}}$, where *T* represents a ‘contrast’ threshold that serves as a soft cut-off to increase the contrast between low and high PAE values. This increased contrast leads to more distinct, better-defined clusters. The threshold *T* can be changed by the user as it is sometimes necessary when working with AlphaFold predictions that may be overly confident due to the inclusion of identical or very similar protein in the training data of the AlphaFold model. The aforementioned MET8 protein illustrates this issue, as shown in Fig. 1A and B, where the pLDDT and PAE values around all but one disordered segment is much better than we would typically expect. By adjusting the contrast threshold, the difference in inter- versus intra-domain PAE values between various structural regions can be more accurately captured, leading to a clearer distinction.

### 2.2 Clustering to segment the protein

The Leiden clustering algorithm is used to partition the weighted protein structure graph into distinct clusters, representing various segments of the protein. This clustering algorithm features a tuneable parameter known as the resolution parameter, which modulates the granularity of the clusters by balancing intra-cluster density against inter-cluster separation. Adjusting this parameter allows for control over the number of clusters: higher resolution values yield a greater number of smaller clusters, while lower values result in fewer, larger clusters (Traag *et al.* 2019). The resulting clusters are subsequently mapped back onto the protein structure, delineating the different structural partitions of the protein (see Fig. 1C).

Recommended parameter settings, based on benchmarking with 1000 randomly selected samples from the CATH and ECOD databases (see Supplementary Information, available as supplementary data at *Bioinformatics* online), are a resolution of 0.7 and a threshold of 2. These values serve as effective starting points for domain segmentation. Benchmarking involved calculating the Intersection over Union (IoU) across the AFragmenter parameter space, with the 10 best parameter combinations yielding mean IoU values of 0.71–0.75 for CATH and 0.71–0.72 for ECOD (see Fig. 1, available as supplementary data at *Bioinformatics* online). For initial exploration, resolution values in the range of 0.4–0.8 and threshold values between 0 and 4 are suggested. Higher values for either parameter tend to yield a larger number of smaller domains, while lower values result in fewer, broader domains. However, depending on the specific application, values outside these ranges may also be informative.

## 3 Discussion

Protein domain segmentation is a complex problem, often with multiple possible solutions. This can be explained by the absence of a standardized definition of what constitutes a protein domain. Our AFragmenter tool tackles two key limitations of existing methods: First, all but one of the tools surveyed return single solutions, and their uses may miss alternative interpretations (see Fig. 1D). Second, these tools are influenced by the specific domain segmentation scheme used by the data source used during training, optimization and evaluation. We suspect that there might be a bias towards protein domains from well-studied organisms, making the performance on proteins of lesser studied organisms less reliable due to the lack of ground truths for validation.

One existing tool that addresses the lack of flexibility is SWORD2 (Cretin *et al.* 2022) which returns multiple solutions through generating protein units and testing different merges. This is an elegant way to circumvent the schema issue, but it does not provide any level of control over the segmentation process. AFragmenter provides this control through the contrast threshold and resolution parameters. This distinction sets AFragmenter apart in terms of the control provided to the user.

There are currently two tools that have used AlphaFold structures in their method design, namely Merizo and DPAM (Zhang *et al.* 2023). Merizo used AlphaFold structures to fine-tune its machine-learning model. DPAM uses PAE values to calculate probabilities of residue pairs belonging to the same domain. However, these PAE derived probabilities only account for one tenth of the final scoring, and much more of the final score is dependent on sequence- and structure-based hits to known domains in ECOD.

The clustering approach implemented within AFragmenter is not entirely unique, as a similar method is used by the ‘AlphaFold predicted Alignment Error’ tool within the ChimeraX protein viewer software for colouring protein structure domains (Meng *et al.* 2023). However, AFragmenter differs by using a soft cut-off for enhanced contrast between low and high PAE values, and uses the more robust Leiden algorithm over the Clauset-Newman-Moor greedy algorithm (Blondel *et al.* 2008, Traag *et al.* 2019). Importantly, AFragmenter was designed for ease of use and does not necessitate a full GUI application like ChimeraX.

In individual cases, we observed that recent segmentation methods such as Chainsaw and Merizo perform well without parameter tuning, making them particularly well suited for processing large datasets without supervision. In contrast, AFragmenter is intended to provide a tuneable method that allows finer control over the segmentation. This user control can be valuable for investigating proteins where conventional domain definitions are ambiguous or where specific research questions necessitate alternative structural interpretations, contexts where a single, pre-defined domain solution may not suffice. Such tunability enables users to generate interpretations aligned with specific biological hypotheses or to investigate domain organizations in less-characterized protein systems.

In summary, AFragmenter offers a flexible and schema-free approach to protein structure segmentation by leveraging AlphaFold’s PAE scores and graph theory. By avoiding reliance on predefined segmentation schemas and incorporating user-adjustable parameters, our method provides researchers with an easy-to-use, exploratory, and tuneable strategy for semi-automatic segmentation of protein structures. This capability is particularly beneficial in research areas where standard domain classification may not fully capture functional or evolutionary nuances, offering a complementary and exploratory perspective to database-driven domain assignment.

## Supplementary Material

btaf588_Supplementary_Data

## Data Availability

The data underlying this article and the benchmarking are available on Github at https://github.com/sverwimp/AFragmenter and on Zenodo at https://doi.org/10.5281/zenodo.17215723.
