# Supplementary material for: AFragmenter: schema-free, tuneable protein domain segmentation for AlphaFold protein structures
Source: Bioinformatics. 2025 Oct 27;41(11):btaf588. doi: 10.1093/bioinformatics/btaf588 (PMC12619643; doi:10.1093/bioinformatics/btaf588)
Supplement: btaf588_Supplementary_Data [file btaf588_supplementary_data.zip › Supplementary Information - Benchmarking.pdf]

Supplementary Information

A Benchmarking of AFragmenter

A1 Benchmarking methodology

To evaluate the performance of AFragmenter, we conducted extensive benchmarking against established protein domain definitions. This section details the datasets used, the sampling and filtering procedures, the parameter space explored, and the evaluation metric employed.

A1.1 Benchmarking Datasets and Preprocessing

Benchmarking was performed using protein domain definitions from two widely recognized structural classification databases: CATH version 4.4 (Knudsen and Wiuf, 2010) and ECOD version 292 (Cheng *et al.*, 2014). For both databases, initial data was retrieved from clusters grouped at 40% sequence identity to minimize redundancy.

CATH Dataset: A total of 1000 unique protein entries were randomly sampled from the CATH database. For each selected entry, its PDB identifier was mapped to its corresponding UniProt identifier. The availability of a full-length AlphaFold protein structure (Jumper *et al.*, 2021) in the AlphaFold Protein Structure Database (AFDB) (Varadi *et al.*, 2022) was then verified. To ensure a meaningful comparison between the PDB-derived domain delineation and the AlphaFold-predicted structure, stringent length filtering was applied: only entries where the residue length difference between the PDB structure and its corresponding AlphaFold model was at most 10% were retained. An additional check ensured that the maximum residue index of the ground-truth domain delineation did not exceed the AlphaFold structure's residue length by more than 10%. Only unique PDB and UniProt identifiers were included in the final CATH benchmarking set. AFragmenter's domain predictions on these AlphaFold structures were then compared against the CATH ground-truth delineations across a comprehensive parameter search space, comprising 165 unique combinations. This space included Threshold values ranging from 0 to 10 (with increments of 1) and Resolution values ranging from 0.1 to 1.5 (with increments of 0.1). All other AFragmenter parameters were kept at their default settings.

ECOD Dataset: For the ECOD database, 500 unique protein entries were randomly sampled using the same methodology as for the CATH dataset. In addition, 500 AlphaFold structures were directly sampled from AFDB v4, with their availability serving as the sole criterion for inclusion. AFragmenter's predictions on these structures were similarly compared against the ECOD domain definitions, employing the same parameter search space as used for the CATH dataset.

A1.2 Evaluation Metric

The accuracy of AFragmenter's domain segmentations was quantified using the Intersection over Union (IoU) metric, referred to as IoU_{cha}. This metric was calculated in a manner similar to the approaches employed by Merizo (Lau *et al.*, 2023) and Chainsaw (Wells *et al.*, 2024), assessing the average agreement between predicted and ground-truth domains for a given protein chain.

For each protein chain, the domain pairing process involved associating each ground-truth domain (T_i, represented as a set of residue indices) from the reference database with at most one predicted domain (P_i) from AFragmenter's output. This pairing was optimized to maximize the sum of individual IoU values, subject to the following constraints:

1. Each ground-truth domain (T_i) can be paired with at most one predicted domain (P_i)
2. Each predicted domain can be assigned at most once.

It is important to note that IoU values were not computed for residues that were labeled as, or predicted to be, non-domain residues. To derive a single final score for the entire protein chain, each domain-level IoU was weighted by the number of residues in the corresponding ground-truth domain. The IoU_{chain} is formally defined as:

$$IoU_{chain} = \sum_{i=1}^{n_{dom}} \frac{|T_i \cap P_i|}{|T_i \cup P_i|} \cdot \frac{|T_i|}{\sum_{j=1}^{n_{dom}} |T_j|}$$

Where n_{dom} denotes the total number of ground-truth domains in the protein chain, $|T_i \cap P_i|$ represents the number of residues in the intersection of ground-truth domain $T_i$and its paired predicted domain P_i, $|T_i \cup P_i|$ signifies the number of residues in their union, and $\sum_{j=1}^{n_{dom}} |T_j|$ corresponds to the total number of residues across all ground-truth domains within that chain.

A1.3 Benchmarking results

To systematically assess the impact of AFragmenter's key parameters on domain segmentation accuracy, a comprehensive grid search was performed across varying Threshold and Resolution values. The results, presented as heatmaps in Figure S1, illustrate the mean IoU values obtained when comparing AFragmenter's predictions against domain definitions from the CATH and ECOD databases.

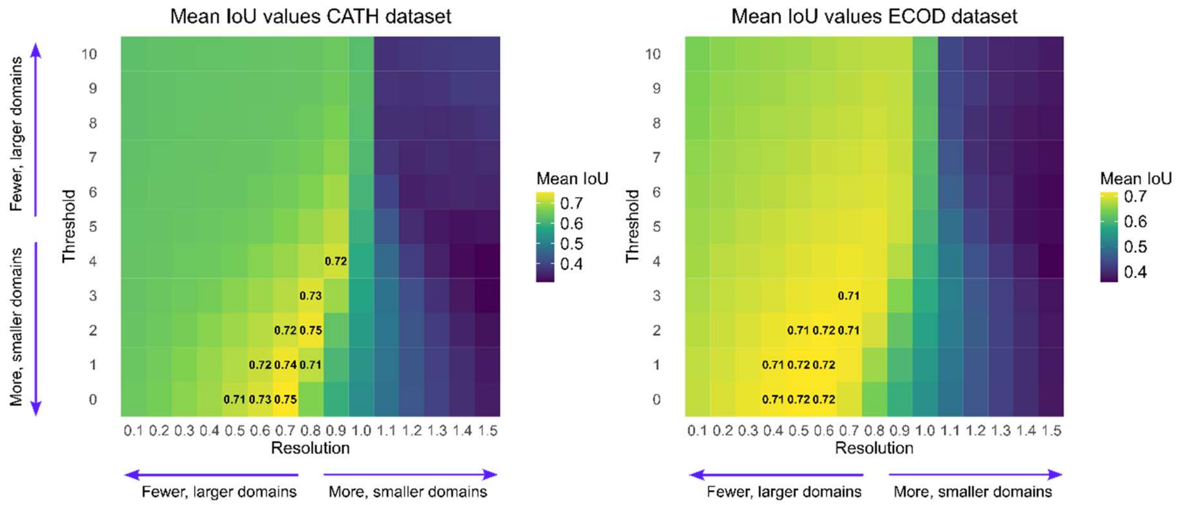

Figure S1: **Mean Intersection over Union (IoU) values for AFragmenter across parameter search space**. The x-axis represents the Resolution parameter (0.1 to 1.5), and the y-axis represents the Threshold parameter (0 to 10). Only the 10 highest mean IoU values are numerically indicated within each plot.

A1.3.1 General Trends and Parameter Influence

Across both CATH and ECOD datasets, consistent trends in AFragmenter's performance are observed over the investigated parameter ranges. Performance significantly declines at Resolution values above approximately 1.0-1.1, regardless of the Threshold, indicating that higher resolution settings consistently lead to over-segmentation. Similarly, Threshold values above approximately 7-8 tend to reduce mean IoU, likely due to overly permissive clustering leading to under-segmentation. Conversely, specific regions of the parameter space consistently yield higher IoU values.

A1.3.2 Dataset-Specific Performance

CATH Dataset: For the CATH dataset, the highest mean IoU values cluster prominently around Thresholds 0-4 and Resolutions 0.5-0.8. The peak performance of **0.75** is observed at Threshold=0 & Resolution=0.7 and also at Threshold=2 & Resolution=0.8, highlighting these settings as particularly effective for matching CATH's domain schema. Other high-performing combinations (e.g., 0.74 at T=1, R=0.7; 0.73 at T=0, R=0.6 and T=3, R=0.8) further define this optimal region for CATH-like domain schemas.

ECOD Dataset: The ECOD dataset generally exhibits a similar optimal region, though with a slightly broader distribution of high IoU values and a marginally lower absolute peak performance. The highest mean IoU achieved for ECOD is **0.72**, observed across several combinations, including Threshold=0 & Resolution=0.6 and Threshold=2 & Resolution=0.6. The overall pattern of high performance remains within Thresholds 0-4 and Resolutions 0.4-0.8. The subtle differences in the distribution of peak performance compared to CATH might reflect the inherent differences in domain granularity or principles between the two databases.

A1.3.3 Recommended Parameter Search Space

Based on these comprehensive benchmarking results, if a user desires domain segmentations that are structurally analogous to those found in established databases like CATH or ECOD, we recommend focusing on the following parameter search space for AFragmenter:

- **Threshold:** 0 to 4
- **Resolution:** 0.4 to 0.8

Within this empirically derived range, users can fine-tune the parameters to explore different levels of domain granularity while achieving good agreement with reference datasets. Specific values like Threshold=1 and Resolution=0.7 (for CATH-like comparisons) or Threshold=0 and Resolution=0.6 (for ECOD-like comparisons) are observed to yield high performance. This recommended search space provides a practical starting point for domain delineation with AFragmenter.

A1.4 Analysis of IoU variability

Beyond mean performance, understanding the distribution and variability of IoU values across different parameter settings is crucial for a comprehensive assessment of AFragmenter. Figures S2 and S3 present violin plots illustrating the full distribution of IoU scores for varying resolution values, keeping the threshold fixed at 2. These figures complement the heatmaps by providing insight into the spread and density of IoU values, rather than just the mean.

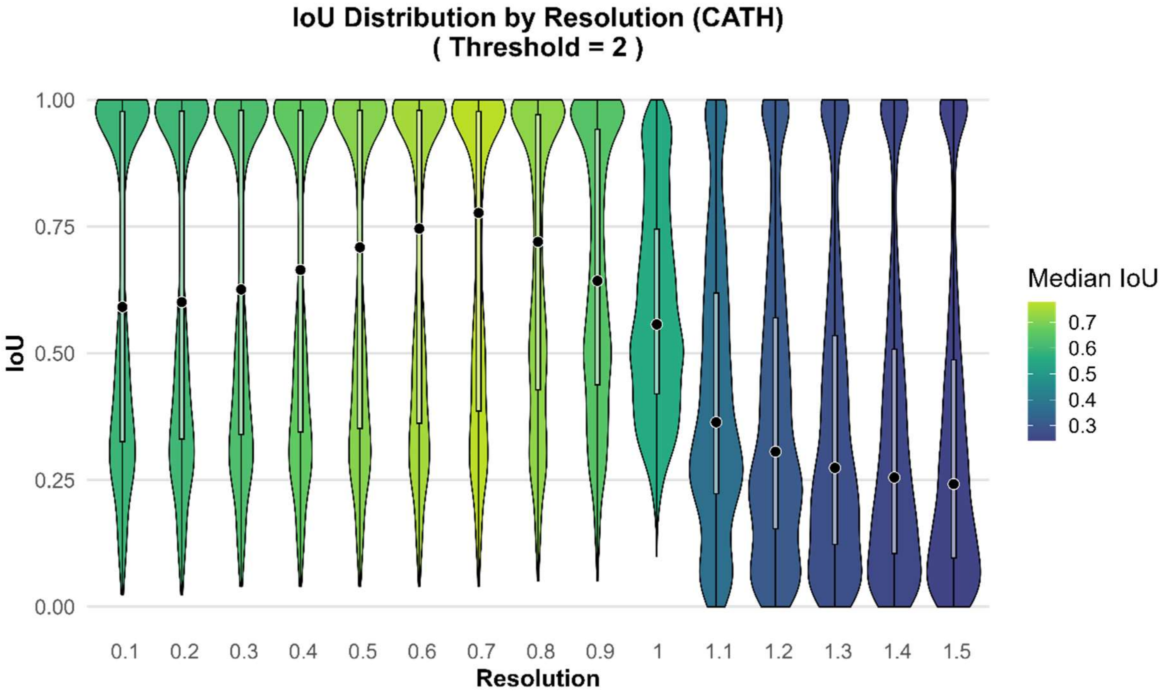

Figure S2: **IoU Distribution by Resolution (CATH, Threshold = 2).** Distribution of IoU values for AFragmenter's segmentations on the CATH dataset. Each violin shows the density distribution of IoU scores for a given resolution, with the black dot representing the median IoU. The violins are coloured by their median IoU, ranging from low (blue) to high (light green).

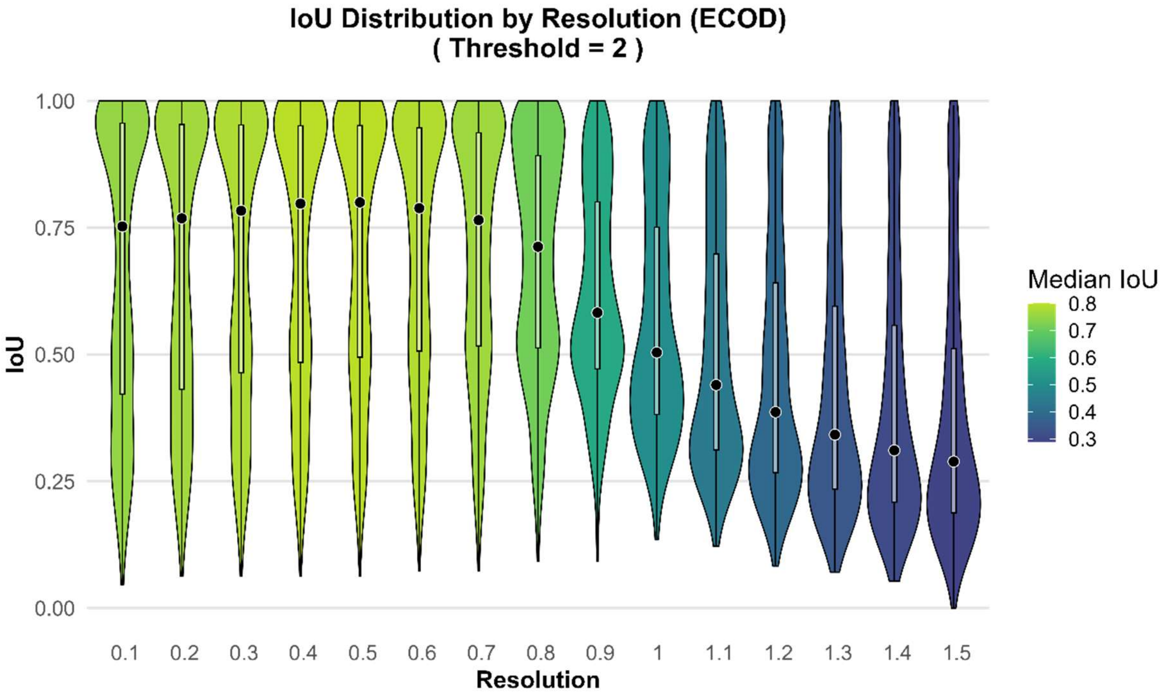

Figure S3: **IoU Distribution by Resolution (ECOD, Threshold = 2).** Distribution of IoU values for AFragmenter's segmentations on the ECOD dataset. Each violin shows the density distribution of

IoU scores for a given resolution, with the black dot representing the median IoU. The violins are coloured by their median IoU, ranging from low (blue) to high (light green).

Figures S2 (CATH) and S3 (ECOD) both demonstrate how the distribution of IoU values changes across the tested resolution range when the threshold is held constant at 2. A general trend observed in both datasets is that median IoU values initially increase with resolution, reach a plateau or peak, and then decline sharply at higher resolutions.

For the **CATH dataset (Figure S2)**, the median IoU peaks around a resolution of 0.7-0.8, with the distributions showing a higher density of scores above 0.7. At resolutions lower than 0.5 (e.g., 0.1-0.4), the median IoU is lower (around 0.6-0.65), and the violins are somewhat wider, indicating a broader spread of performance. As resolution increases beyond 0.9, the distributions become heavily skewed towards lower IoU values (below 0.4), signifying a consistent tendency towards over-segmentation.

Similarly, for the **ECOD dataset (Figure S3)**, the highest median IoU values are observed in the resolution range of approximately 0.4 to 0.8. Notably, ECOD tends to maintain slightly higher median IoU values at very low resolutions (e.g., 0.1, 0.2) compared to CATH. However, like CATH, the performance for ECOD also deteriorates significantly at higher resolutions (above 1.0), with IoU distributions concentrating at very low values, albeit with a slightly less abrupt decline than observed for CATH.

In both cases, the width and shape of the violins highlight the variability of AFragmenter's performance. In the optimal resolution ranges, while median IoU is high, there is still a considerable spread of values, indicating that even within optimal parameters, segmentation accuracy can vary depending on the specific protein architecture. At resolutions leading to over-segmentation (e.g., ≥ 1.0), the distributions become highly concentrated at low IoU values, demonstrating a systematic bias towards poor agreement with reference databases under these conditions. This detailed distributional analysis confirms that careful parameter selection, particularly for resolution, is crucial for achieving reliable domain predictions that align with established classifications.

A1.5 Concluding remarks

The comprehensive benchmarking presented in this section evaluates AFragmenter's performance in delineating protein domains against classifications from CATH and ECOD. Our analysis indicates the significant influence of both the threshold and resolution parameters on segmentation accuracy, identifying specific parameter ranges that yield high agreement with these reference databases. Performance across diverse protein architectures, as evidenced by IoU distributions, highlights the inherent variability in domain definitions. This underscores AFragmenter's design as a parameter-driven method, which leverages AlphaFold's predicted aligned error (PAE) to provide users control over segmentation granularity. Through the exploration of empirically defined parameter spaces, AFragmenter offers the flexibility to generate multiple, consistent domain solutions, thereby enabling researchers to tailor predictions to specific structural contexts or explore alternative domain definitions, supporting its application in diverse domain annotation tasks.

References

Cheng,H. *et al*. (2014) ECOD: An Evolutionary Classification of Protein Domains. *PLoS Comput. Biol.*, **10**, e1003926.

Jumper,J. *et al*. (2021) Highly accurate protein structure prediction with AlphaFold. *Nature*, **596**, 583–589.

Knudsen,M. and Wiuf,C. (2010) The CATH database. *Hum. Genomics*, **4**, 207–212.

Lau,A.M. *et al*. (2023) Merizo: a rapid and accurate protein domain segmentation method using invariant point attention. *Nat. Commun.*, **14**, 8445.

Varadi,M. *et al*. (2022) AlphaFold Protein Structure Database: massively expanding the structural coverage of protein-sequence space with high-accuracy models. *Nucleic Acids Res.*, **50**, D439–D444.

Wells,J. *et al*. (2024) Chainsaw: protein domain segmentation with fully convolutional neural networks. *Bioinformatics*, **40**, btae296.
